# Analysis of self-transcendence status and influencing factors in gastric cancer patients undergoing chemotherapy: a random forest model-based study

**DOI:** 10.3389/fpsyt.2025.1641453

**Published:** 2025-09-15

**Authors:** Qin Wang, Guoqin Ren, Li Sun, Xumiao Zhang, Hongxia Hua, Yanglin Gu

**Affiliations:** ^1^ Wuxi School of Medicine, Jiangnan University, Wuxi, China; ^2^ Department Public Health, Jiangnan University Medical Center, Wuxi, China; ^3^ Department Oncology, Jiangnan University Medical Center, Wuxi, China; ^4^ Department Orthopedic, Jiangnan University Medical Center, Wuxi, China

**Keywords:** gastric cancer, chemotherapy, self-transcendence, random forest model, influencing factors, artificial intelligence

## Abstract

**Objective:**

To investigate the current status and determinants of self-transcendence in gastric cancer patients undergoing chemotherapy and to establish a foundation for clinical development of interventions.

**Methods:**

A convenience sampling method was employed to select 507 gastric cancer patients undergoing chemotherapy in the oncology department of a tertiary hospital in Wuxi City, Jiangsu Province, from October 2024 to May 2025. Questionnaire surveys were carried out using the Demographic Characteristics Questionnaire, Brief Illness Perception Questionnaire, Herth Hope Index, Medical Coping Modes Questionnaire, and Self-Transcendence Scale. A random forest model and LASSO regression were used to rank the importance of influencing factors and select characteristic variables. Then, multiple linear regression analyses were combined to determine the main influencing factors.

**Results:**

The mean value of the self-transcendence score of gastric cancer patients undergoing chemotherapy was 44.08. The random forest model results demonstrated that prioritizing the key variables was most effective at a lambda (λ) value of 0.048, which aligned with five influencing factors. The independent variables with the top five importance rankings were the degree of confrontation, the self-rated health status, the degree of resignation, the level of hope, and the degree of avoidance. Multiple linear regression analysis revealed that self-transcendence in gastric cancer patients undergoing chemotherapy was predominantly affected by confrontation degree, self-rated health status, resignation degree, hope level, and avoidance degree (*P*<0.05).

**Conclusion:**

The self-transcendence level of gastric cancer patients undergoing chemotherapy is observed to be in the moderate to low spectrum. Healthcare personnel can improve this level by targeting the relevant influencing factors, thereby enhancing quality of life during chemotherapy.

## 1 Introduction

Gastric cancer is a prevalent malignant tumor and a leading cause of cancer-related mortality globally. According to the latest data (1) released by the International Agency for Research on Cancer (IARC), over 968,000 new gastric cancer cases and approximately 660,000 deaths were reported worldwide in 2022, ranking it as the fifth most common cancer and cause of cancer-related death, characterized by its high disease burden, rapid progression, and elevated mortality (2). The global five-year survival rate remains approximately 20% (3). For patients diagnosed with advanced gastric or inoperable gastric cancer that is not amenable to surgical treatment, fluoropyrimidine and platinum-based medications are commonly used for chemotherapy (4). The approach to chemotherapy differs between Eastern and Western countries (3). In Eastern nations, patients with gastric cancer generally have D2 lymph node dissection (LND), succeeded by adjuvant chemotherapy. In Western nations, perioperative chemotherapy is predominantly employed, either in conjunction with radiotherapy (CRT) or as adjuvant chemotherapy. Prior research indicates that gastrointestinal malignancies might readily induce psychological troubles via the bidirectional link between the microbiome-gut-brain axis and psychological diseases, including depression (5). During the lengthy course of chemotherapy, 63% of gastric cancer patients undergoing chemotherapy have experienced psychological stress such as despair, anxiety, or maladaptive avoidance (6). A healthy psychological state lowers the risk of treatment interruption, reduces anxiety and depression symptoms, encourages adaptation to the disease role, and improves quality of life. It is necessary to improve the psychological well-being of patients with gastric cancer.

Self-transcendence (7) is a psychological construct that reflects an individual’s capacity to transcend stressful life events through psychosocial and spiritual growth. The general population’s pursuit of self-transcendence arises from the active exploration of needs, whereas cancer patients undergo adaptive transformation driven by the urgent necessity of survival. It empowers patients to achieve heightened well-being and life satisfaction when confronting existential challenges, serving as a pivotal predictor of quality of life in oncology patients (8). Self-transcendence theory posits that individuals may improve their psychological resilience by overcoming the constraints associated with their illnesses. Self-transcendence theory comprises four dimensions (9): individual-environment interaction, temporal integration, intrapersonal deepening, interpersonal expansion, and spiritual connectedness. Elevated self-transcendence enables individuals to diminish excessive self-preoccupation while strengthening interpersonal and environmental connections, thereby enhancing subjective well-being and psychological health (10). By developing this ability in patients, individuals are able to mobilize resources in crisis, rebuild psychological resilience, and rediscover the purpose and meaning of life (11), as well as enhance self-efficacy, reduce anxiety and depression, and improve overall quality of life. Maslow’s hierarchy of needs theory states that cancer patients during treatment typically only have access to fundamental safety and physiological requirements. However, the attainment of self-transcendence allows these people to feel higher degrees of fulfillment, such as love and belonging, self-esteem, and eventually self-actualization, that are comparable to those experienced by the healthy population (12).

Existing studies indicate that cancer patients generally exhibit low-to-moderate self-transcendence levels (13). However, current research predominantly focuses on breast and lung cancers, with fewer investigations on the current status and influencing factors of self-transcendence in gastric cancer patients undergoing chemotherapy. Self-transcendence as a psychosocial variable may have complex nonlinear relationships and interactions. The absence of machine learning-based quantitative analysis in current research makes it challenging to capture the intricate interactions between variables (14). Responding to these deficiencies, this study employs a random forest model to investigate the current status and predictors of self-transcendence in patients with gastric cancer undergoing chemotherapy (15). Variable importance ranking and LASSO regression analysis are integrated to identify key determinants, aiming to establish an evidence-based foundation for developing targeted clinical interventions to enhance self-transcendence in this vulnerable population (16).

## 2 Methods

### 2.1 Study setting and sampling

We used the convenience sampling method to select patients. A total of 524 patients with gastric cancer who received chemotherapy were recruited from a tertiary Grade A hospital in Wuxi, China, between October 2024 and May 2025. Based on the heuristic method for quantitative studies (17), the minimum required sample size was determined as 5~10 times the number of independent variables. With 31 predictor dimensions in the study scale, the target range was 155~310 participants. Accounting for a 10% anticipated invalid response rate, and combined with the opinions of clinical experts, the actual collection of questionnaires was 524 cases. Inclusion criteria: (1) Confirmed diagnosis of gastric cancer through histopathological and imaging examinations; (2) The department head assesses the expected survival time to be greater than 6 months based on the gastric cancer diagnosis and treatment guidelines; (3) Aged ≥18 years with literacy skills to complete questionnaires reliably; (4) Voluntary participation with informed consent. Exclusion criteria: (1) Comorbid cognitive impairment, hearing/language deficits, or psychiatric disorders; (2) Physical frailty precluding questionnaire completion.

### 2.2 Instruments

#### 2.2.1 Demographic Characteristics Questionnaire

This self-designed questionnaire comprised two sections: (1) Sociodemographic data, including age, gender, educational attainment, marital status, occupation, monthly income, religious faith, living arrangements, and smoking and alcohol consumption. (2) Clinical data: Treatment modalities, comorbidities, disease duration, and self-rated health status.

#### 2.2.2 Brief Illness Perception Questionnaire

The questionnaire was developed by Broadbent et al. (18). This validated tool quantifies patients’ illness perceptions across cognitive, emotional, and comprehension domains. The 9-item scale includes eight Likert-type items (0~10 points) and one open-ended question. Items 3, 4, and 7 are reverse-scored, yielding a total score of 0~80 (excluding the open response). Higher scores indicate stronger negative perceptions of disease threat. The Cronbach’s α in this study was 0.82, demonstrating good reliability and validity.

#### 2.2.3 Herth Hope Index

The questionnaire was developed by Herth (19). This 12-item scale assesses hope levels in cancer patients through three dimensions: positive attitudes toward reality and the future, proactive behaviors, and interpersonal connectedness. Items are rated on a 4-point Likert scale (1 = “strongly disagree” to 4 = “strongly agree”), with items 3 and 6 reverse-scored. Total scores classify hope levels as low (12~23), moderate (24~35), or high (36~48). The Cronbach’s α in this study was 0.85, demonstrating good reliability and validity.

#### 2.2.4 Medical Coping Modes Questionnaire

The questionnaire was developed by Feifel et al. (20) consisted of 19 items. Chinese scholars Shen and Jiang (21) completed the systematic revision of the Chinese version, comprising 20 items with three subscales: confrontation (8 items), resignation (5 items), and avoidance (7 items). Eight items are reverse-scored using a 4-point Likert scale, widely applied in Chinese clinical settings. The overall score is 20 points. The higher the score in each area, the more inclined one is toward the related coping style. The Cronbach’s α in this study was 0.80, demonstrating good reliability and validity.

#### 2.2.5 Chinese version of the Self-Transcendence Scale

The questionnaire was adapted from Reed (22)’s 15-item unidimensional scale by Zhang et al. (23) in 2014. This questionnaire measures self-transcendence using a 4-point Likert scale, with a total score ranging from 15 to 60. 1 to 4 points respectively represent “not at all,” “only a little,” “some,” and “a lot”. Scores ≤45 indicate low self-transcendence, while scores>45 reflect high levels. Higher scores denote greater self-transcendence capacity. The Cronbach’s α in this study was 0.88.

### 2.3 Data collection

Two trained nursing postgraduate students collected data using paper questionnaires with institutional consent. Researchers obtained written informed permission from eligible participants after explaining the study purpose, procedures, and confidentiality protocols. Participants independently completed the questionnaires. To assure response accuracy, researchers gave standardized verbal help in face-to-face interviews for persons with literacy or physical limitations. The entire process required approximately 10~30 minutes per participant. Completed questionnaires were immediately reviewed on-site to detect and rectify discrepancies or missing entries. Post-collection verification included rigorous checks for completeness, with exclusion criteria applied to questionnaires that contained more than 10% missing data. A total of 524 questionnaires were distributed, with 17 excluded due to incompleteness or ineligibility, resulting in a valid response rate of 96.76%.

### 2.4 Data analysis

Data were analyzed using SPSS 27.0 and R Studio. Descriptive statistics included frequency and percentage for categorical variables and mean ± standard deviation for continuous variables. Independent-samples t-tests or one-way ANOVA were used to compare differences in self-transcendence scores across demographic subgroups. Pearson’s correlation analysis identified associations between key variables. Variables showing statistical significance (*P* < 0.05) in univariate or correlation analyses were entered into a random forest model (RStudio) for variable importance ranking. LASSO regression analysis facilitated variable selection, followed by multiple linear regression analysis to determine relationships between self-transcendence and predictors such as hope levels. Statistical significance was set at *P* < 0.05.

### 2.5 Ethical considerations

This study obtained ethical approval from the Institutional Review Board of the participating tertiary hospital (Approval No: Y-305, 2024). The investigation strictly adhered to ethical principles of voluntary participation, justice, confidentiality, and beneficence/non-maleficence. Data collection protocols ensured anonymity through coded identifiers, with all records stored in password-protected systems accessible only to the research team.

## 3 Results

### 3.1 Socio-demographic characteristics and univariate analysis

This study comprised 507 participants, with a mean age of 68.62 years (SD = 10.07). The sample consisted of 181(35.7%) with higher education, 361(71.2%) married, and 472(93.1%) covered by health insurance. 237(46.7%) seldom participated in group activities, and 432(85.2%) considered their health to be poor. After t-test and ANOVA analysis, the outcomes showed that the dependent variable revealed statistically significant associations (*P* < 0.05) between self-transcendence levels and the following variables: age, education, marital status, occupation, monthly income, religious affiliation, living arrangements, Group Activity Participation, personality traits, payment method, number of comorbidities, and self-rated health status. Details are shown in **Table 1**.

**Table 1:** Social-demographic characteristics of participants and comparison of different variables on self-transcendence (N = 507).

Variable	Category	Frequency (n)	Percentage (%)	Mean	SD	*F*/*t*	*P*
Gender	Male	297	58.6	1.41	0.493	0.902	0.368
	Female	210	41.1				
Age (years)	18-60	99	19.5	2.35	0.87	5.75	0.001^a^
	60-70	167	32.9				
	70-80	206	40.6				
	≥80	35	6.9				
Education	Primary school or below	108	21.3	2.22	0.868	2.846	0.037b
	Junior high school	218	43				
	Senior high school	142	28				
	College or above	39	7.7				
Marital Status	Single	2	0.4	2.47	0.796	5.096	0.002^a^
	Married	361	71.2				
	Divorced	49	9.7				
	Widowed	95	18.7				
Occupation	Farmer	127	25	2.26	1	5.442	0.001^a^
	Worker	195	38.5				
	Public institution	112	22.1				
	Corporate employee	71	14				
	Other	2	0.4				
Monthly Income (CNY)	≤2000	116	22.9	2.36	0.997	7.491	0.001^a^
	2001~4000	168	33.1				
	4001~6000	146	28.8				
	>6000	77	15.2				
Religious faith	Yes	83	16.4	1.84	0.37	t3.881	0.001^a^
	No	424	83.6				
Living Arrangements	Living alone	53	10.5	2.96	1.042	6.493	0.001^a^
	spouse	116	22.9				
	children	149	29.4				
	spouse and children	178	35.1				
	relatives/friends	11	2.2				
Group Activity Participation	Yes	270	53.3	1.47	0.499	2.895	0.004^a^
	No	237	46.7				
Personality Traits	Extroverted	287	56.6	1.43	0.496	5.778	0.001^a^
	Introverted	220	43.4				
Smoking	Yes	110	21.7	2.040	0.688	0.099	0.906
	No	267	52.7				
	Have given up	130	25.6				
Drinking	Yes	102	20.1	2.04	0.664	2.088	0.125
	No	283	55.8				
	Have given up	122	24.1				
Payment method	Employee insurance	305	60.2	1.51	0.74	12.228	0.001^a^
	Resident insurance	167	32.9				
	Out-of-pocket	15	3				
	Other	20	3.9				
Major event	Yes	243	47.9	1.52	0.5	-0.074	0.941
	No	264	52.1				
First Consultation time (years)	≤1	292	57.6	1.49	0.617	4.348	0.013
	1 ~5	182	35.9				
	≥5	33	6.5				
Chronic	0	127	25	2.19	0.898	0.973	0.405
	1	195	38.5				
	2	147	29				
	≥3	49	9.7				
Family history	Yes	90	17.8	1.82	0.382	-1.42	0.156
	No	419	82.2				
Complications	0~1	203	40	1.79	0.739	26.936	0.001^a^
	2~3	208	41				
	>3	96	18.9				
Treatment programme	operation	127	25	2.14	0.789	1.558	0.212
	chemotherapy	182	35.9				
	operation + chemotherapy	198	39.1				
Self-Rated health status	Good	75	14.8	3.14	0.684	35.415	0.001^a^
	Fair	299	59				
	Poor	120	23.7				
	Very poor	13	2.6				
Disease stage	≤II	139	27.4	1.9	0.662	25.232	0.001^a^
	III	280	55.2				
	≥IV	88	17.4				
Metastasis status	Yes	315	62.1	1.38	0.486	-5.913	0.001^a^

a P < 0.01, P < 0.05.

### 3.2 Analysis of the current status and correlation of illness perception, hope levels, medical coping modes, and self-transcendence among gastric cancer patients undergoing chemotherapy

The assessment outcomes revealed the following scores (presented as mean ± SD): The mean score for illness perception was 42.79 (SD = 9.30); The mean score for Hope Levels was 34.10 (SD = 9.21); The mean score for confrontation was 24.50 (SD = 6.51); The mean score for Avoidance subscale was 20.69 (SD = 6.30); The mean score for Resignation subscale was 14.70 (SD = 4.61); The mean score for Self-Transcendence was 44.08 (SD = 10.38). The findings demonstrated significant correlations between self-transcendence levels and key psychosocial variables. Self-Transcendence is positively correlated with Hope Levels (r =0.579, *P* < 0.01) and Confrontation (r =0.517, *P* < 0.01). Self-Transcendence is negatively correlated with illness perception (r =-0.545, *P* < 0.01), Avoidance (r =-0.517, *P* < 0.01), and Resignation (r =-0.530, *P* < 0.01). The correlation analysis of other variables is shown in **Table 2**.

**Table 2:** Correlation analysis between self-transcendence levels and influencing factors.

Variable	1	2	3	4	5	6	7	8	9	10	11	12
1	1												
2	.540**	1											
3	.493**	.415**	1										
4	.947**	.753**	.640**	1									
5	-.411**	-.385**	-.374**	-.468**	1								
6	-.358**	-.385**	-.349**	-.427**	.610**	1							
7	-.421**	-.380**	-.405**	-.479**	.582**	.595**	1						
8	-.464**	-.448**	-.439**	-.535**	.859**	.861**	.845**	1				
9	.401**	.423**	.376**	.473**	-.462**	-.426**	-.487**	-.536**	1			
10	-.384**	-.387**	-.378**	-.450**	.488**	.454**	.391**	.521**	-.304**	1		
11	.443**	.421**	.362**	.500**	-.448**	-.452**	-.493**	-.543**	.714**	-.333**	1		
12	-.462**	-.474**	-.454**	-.545**	.487**	.504**	.494**	.579**	-.517**	.517**	-.530**	1	

**At the 0.01 level (two-tailed), the correlation is significant.

1 Cognitive dimensions, 2 Emotional dimensions, 3 Comprehension dimensions, 4 Illness Perception, 5 Positive attitudes toward reality and the future, 6 Taking positive action, 7 Maintaining close contact with others, 8 Hope levels, 9 Avoidance, 10 Confrontation, 11 Resignation, 12 Self-transcendence.

### 3.3 Screening of influencing factors for self-transcendence in gastric cancer patients undergoing chemotherapy

#### 3.3.1 Variable importance ranking

**Figure 1:**
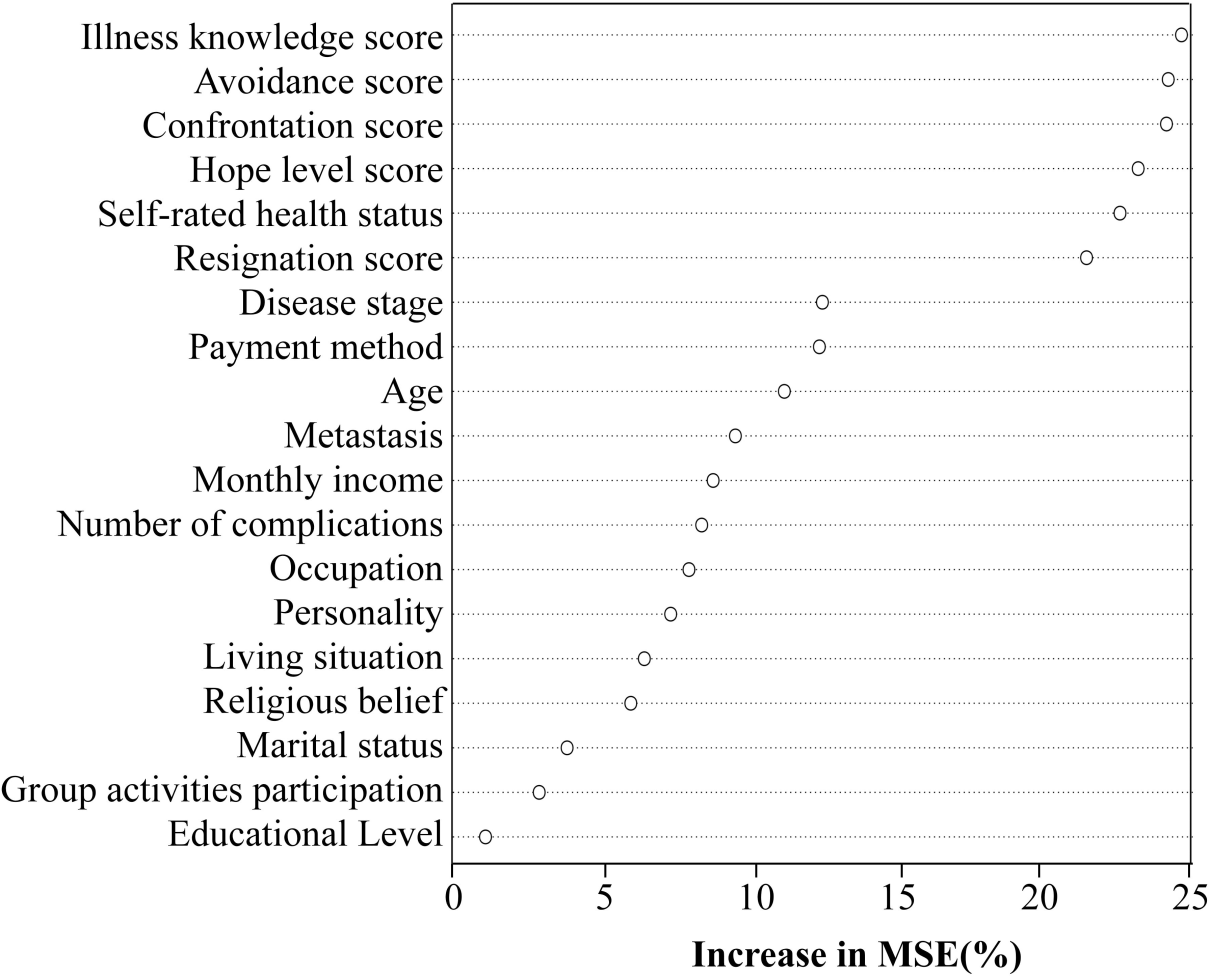
Variable importance ranking of influencing factors for self-transcendence in gastric cancer patients undergoing chemotherapy.

A random forest model was constructed in R Studio using self-transcendence levels as the dependent variable. Nineteen variables identified as statistically significant in univariate and correlation analyses were coded and incorporated into the model, with coding protocols detailed in **Table 3** Variable importance was assessed via the percentage increase in mean squared error (%Inc MSE), where higher values indicate greater predictive importance. (24) The analysis revealed the following descending order of variable importance: Illness perception level, Avoidance, Confrontation, Hope levels, Self-rated health status, Resignation, which are shown in **Figure 1**.

**Table 3:** Variable coding in the random forest model.

Variable	Assignment method
Age (years)	18~59 = 1,60~69 = 2,70~79 = 3, >80 = 4
Education	Primary school or below=1, Junior high school=2, Senior high school=3, College or above=4
Marital Status	Single=1, Married=2, Divorced=3, Widowed=4
Occupation	Farmer=1, Worker=2, Public institution=3, Corporate employee=4, Other=5
Monthly Oncome (CNY)	<2000 = 1, 2001~4000 = 2, 4001~6000 = 3, >6000 = 4
Religious faith	Yes=1, No=2
Living Arrangements	Living alone=1, With spouse=2, With children=3, With spouse and children=4, Relatives/friends=5
Group Activity Participation	Yes=1, No=2
Personality Traits	Extroverted=1, Introverted=2
Payment Method	employee insurance=1, resident insurance=2, Out-of-pocket=3, Other=4
Complications	0~1 = 1, 2~3 = 2, >3 = 3
Self-Rated Health Status	Perfect=1, Good=2, Fair=3, Poor=4, Very poor=5
Disease Stage	<II=1, III=2, ≥IV=3
Metastasis Status	Yes=1, No=2
Illness Perception	Original value input
Hope levels	Original value input
Confrontation	Original value input
Avoidance	Original value input
Resignation	Original value input

#### 3.3.2 Variable selection

**Figure 2:**
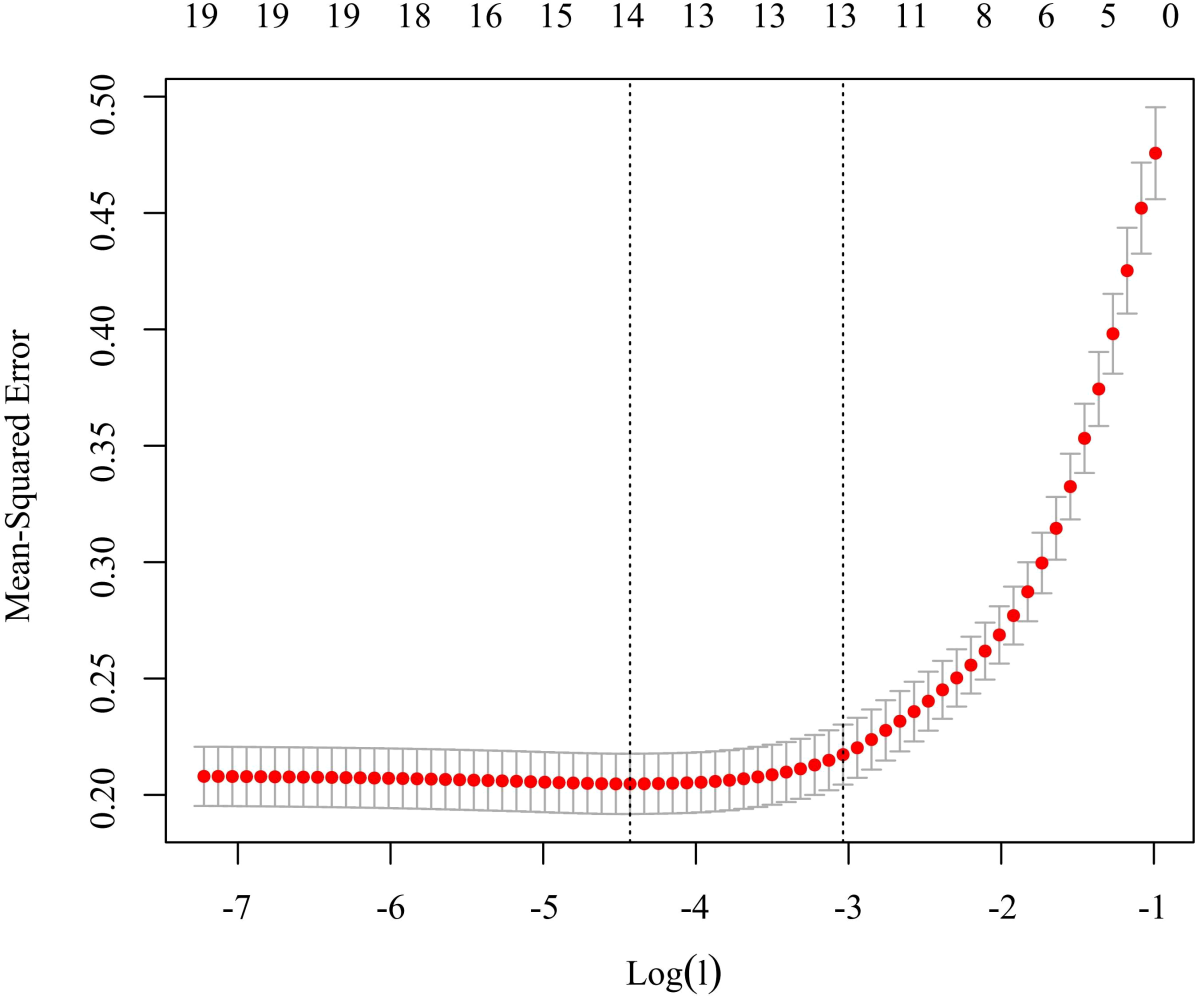
Feature Selection via LASSO Regression Analysis.

LASSO regression was performed using the glmnet package in R Studio to refine the 19 statistically significant variables. At a lambda (λ) value of 0.048 (tuning parameter), the model achieved optimal parsimony with minimal prediction error, retaining five key predictors: confrontation coping, self-rated health status, resignation coping, hope levels, and avoidance coping. These variables were subsequently entered into multivariate stepwise regression analysis. Details are shown in **Figure 2**.

### 3.4 Multivariate analysis of influencing factors for self-transcendence in gastric cancer patients undergoing chemotherapy

A multivariate stepwise linear regression analysis was conducted with the total self-transcendence score as the dependent variable. The top five predictors identified through LASSO and random forest analyses were included as independent variables. Results demonstrated that confrontation coping, self-rated health status, resignation coping, hope levels, and avoidance coping were significant determinants of self-transcendence (*P* < 0.05). Details are shown in **Table 4**.

**Table 4:** Multivariate stepwise linear regression results for self-transcendence influencing factors.

Variable	B	SE	β	t	*P*	VIF
Constant	48.528	3.216	–	15.091	0.001^a^	–
Confrontation	3.262	0.460	0.256	7.097	0.001^a^	1.386
Self-rated health status	-3.860	0.480	-0.255	-8.043	0.001^a^	1.069
Resignation	-2.178	0.512	-0.194	-4.252	0.001^a^	2.214
Hope levels	0.236	0.047	0.210	5.031	0.001^a^	1.856
Avoidance	-1.583	0.524	-0.137	-3.024	0.003^a^	2.200

β, standardized coefficient β; B, unstandardized coefficient; SE, standard error of B; VIF, variance inflation factor.

a *P* < 0.01.

## 4 Discussion

### 4.1 Demographic factors

The mean self-transcendence score of the cohort was 44.08 (SD = 10.38), with scores ≤45 categorized as low-level self-transcendence (22). This indicates a predominant low level of self-transcendence among gastric cancer patients receiving chemotherapy However, compared with Bozkurt and Yildirim (11)’s research, the self-transcendence levels of gastric cancer patients undergoing chemotherapy have improved, the self-transcendence score in this research was 35.80 (SD = 4.36). The random forest model results indicate that among the ten most significant factors affecting self-transcendence levels, demographic factors comprise self-rated health status, disease stage, medical insurance payment method, age, and the presence of metastases. Firstly, patients’ self-rated health status is a critical determinant affecting levels of self-transcendence. In this study, more than half of the patients (59.0%) assessed their health status as average, which partially accounts for the somewhat elevated self-transcendence scores compared to prior studies. According to Reed’s self-transcendence theory (25), inadequate self-rated health status directly intensifies patients’ “perceived vulnerability.” Chemotherapy-induced physical decline intensifies feelings of bodily disempowerment, resulting in profound existential anxiety (fear of death, sense of meaninglessness), whereas self-transcendence acts as a psychological resource to mitigate this anxiety (26). When patients assess their health status as improved, their self-efficacy increases, facilitating the development of positive intrinsic motivation and fostering spiritual self-transcendence (27). Furthermore, studies reveal that 70% of patients are diagnosed with gastric cancer at an advanced stage (28). In this study, merely 27.4% of patients received a diagnosis at stage II or below. Patients in advanced stages experience a stronger sense of mortality, which can impede the capacity for self-transcendence, preventing it from attaining a significant degree. Thirdly, the extensive adoption of national medical insurance policies has led to a significant percentage of patients in this study qualifying for medical insurance payment (93.1%). The medical insurance payment system mitigates patients’ financial burden, alleviates stress during treatment, and consequently enhances their psychological well-being. Research by Fusco et al. (29) confirmed that medical insurance payment systems influence patients’ degrees of self-transcendence. Fourth, Haugan et al. (30)’s research demonstrated that elderly patients are more likely to attain self-transcendence. The mean age of patients in this study was 68.62 years. Older patients may have a higher level of acceptance of death in terms of cognition and emotion, leading to elevated levels of self-transcendence compared to earlier studies. Fifth, more than half of the patients exhibited metastases (62.1%). Individuals diagnosed with metastatic gastric cancer generally exhibit diminished survival rates and suboptimal treatment outcomes, resulting in increased despondency regarding their prognosis during treatment and making it difficult to achieve high levels of self-transcendence. Consequently, healthcare providers ought to assess self-transcendence levels in patients with adverse health perceptions, advanced disease stages, financial constraints, or metastasis, swiftly recognize and address negative emotions, and support them in enhancing quality of life and health outcomes.

### 4.2 Influencing factors

#### 4.2.1 Impact of illness perception on self-transcendence

The illness perception of gastric cancer patients undergoing chemotherapy was 42.79 (SD = 9.30), indicating a moderate degree. in accordance with Liu et al. (31)’s research. This study identified a negative correlation between illness perception and self-transcendence, which contrasts with the findings by Zhu et al. (32). This discrepancy may stem from divergent measurement frameworks: in our study, higher BIPQ scores reflect stronger negative illness appraisals, whereas Zhu’s instrument equated higher scores with sufficient disease knowledge. Both studies emphasize the importance of correctly perceiving the disease. The physiological and social functioning of gastric cancer patients undergoing chemotherapy is significantly altered by nutritional deficiencies, loss of digestive function, and social isolation, which forces patients to rethink their understanding of the disease and the meaning of life. This leads to a strong bio-psychological coupling effect when patients realize become aware of the fragility of life, which may be comparable to a pursuit of self-transcendent concepts like “spiritual connection” and “meaning of life.” This is consistent with Reed’s theory of self-transcendence, which holds that “insightful transcendence may erupt when a crisis reaches a critical point.” Negative cognition can exacerbate the fear of disease progression in gastric cancer patients (32), reducing their motivation to overcome the disease and preventing them from achieving self-transcendence and overcoming survival anxiety. Healthcare personnel can help patients with gastric cancer improve their illness perception by using cognitive-behavioral therapy (33), delivering disease-specific education on chemotherapy mechanisms and side-effect management, and distribute evidence-based symptom self-care manuals to reduce uncertainty (34).

#### 4.2.2 Impact of hope levels on self-transcendence

The hope level among gastric cancer patients undergoing chemotherapy was 34.10 (SD = 9.21), indicating a moderate level of hope. The level of hope positively predicted self-transcendence, consistent with studies of Er et al. (35) and Li et al. (36). As a psychodynamic catalyst, hope can activate patients’ inner growth potential and continually motivate patients to discover meaning and purpose in life aligns closely with the fundamental principle of self-transcendence, therefore fostering their spiritual development (33). Patients with high hope levels have strong treatment compliance (37), be able to completely mobilize their subjective initiative to face their condition, and regulate their emotions correctly (38). This will help patients complete their transition to the role of patient, achieve psychological growth, and realize self-transcendence. Xue Yang’s research demonstrates that hope levels significantly influence self-transcendence levels in cancer patients (P < 0.05) (39). Thornton et al. conducted a treatment program involving mindfulness, hope therapy, and biobehavioral therapy for 32 women diagnosed with gynecological cancer. Results from a repeated measures design indicated significant reductions in pain, anxiety, and negative emotions during treatment, alongside enhancements in positive emotions and mental health-related quality of life. Additionally, there was a linear increase in self-transcendence and hope levels (P < 0.05) (40). Yang et al. (41)consider psychological intervention a pharmacological research direction for enhancing cancer outcomes, identifying it as an area that requires urgent further development. Mindfulness-based psychological interventions have been shown to effectively enhance psychological conditions, including low hope and anxiety. However, the total number of cancer patients currently undergoing psychological intervention is not yet determined. Healthcare professionals can assist patients in setting realistic, incremental goals via mindfulness interventions (42) or hope therapy (43), or encourage recovered patients to share their experiences to elevate hope levels and promote mental health. This process involves the ‘Goal anchoring -Meaning attribution - Behavioral activation’ chain framework to strengthen psychological resilience, recognize their strengths in vulnerability, and ultimately achieve self-transcendence.

#### 4.2.3 Impact of coping modes on self-transcendence

The confrontation dimension score for gastric cancer patients undergoing chemotherapy for was 24.50 (SD = 6.51), the avoidance dimension score was 20.69 (SD = 6.30), and the resignation dimension score was 14.70 (SD = 4.61). The scores for each dimension exceeded those reported in Ma et al. (44)’s research. This may be due to differences in coping strategies resulting from different disease types. Gastric cancer is often diagnosed at an advanced stage, accompanied by significant uncertainty regarding recurrence and metastasis. Additionally, the subjects measured in this study were chemotherapy patients, whose psychological conditions were more complex, resulting in elevated scores across all three dimensions. However, Cross-cultural analysis reveals that Eastern patients demonstrate greater emotional restraint than their Western counterparts, manifested as higher avoidance/resignation scores and lower confrontation scores. The confrontation dimension scores in this study were also elevated, which may be attributed to the subjective nature of the scale potentially introducing bias in the research results. Confrontation coping positively influenced self-transcendence, while avoidance and resignation exerted adverse effects, aligning with the findings of Mccarthy et al. (45) and Liu et al. (46) Patients with high confrontation scores tended to actively learn about their gastric cancer stage, treatment options, and prognosis. They also engage actively in treatment decision-making and have a sense of control over their disease, which reduces their fear of the unknown. Confrontational coping is often accompanied by healthy behaviors, such as a regular diet and nutritional management, which improve health and contribute to self-transcendence. Avoidance and resignation may increase patients’ negative emotional experiences (47). Patients with a high avoidance score tend to avoid disease-related topics and delay seeking medical treatment, which can lead to disease progression. This reinforces their fear of cancer recurrence (48), hinders their ability to articulate their needs, and can lead to a lack of social support. It can also lead to maintaining the illusion of good health through avoidance and rejecting the patient’s identity, making it challenging to promote self-transcendence. Patients with a high resignation dimension tend to passively accept the disease, giving up their right to fight for their health by believing treatment is ineffective. This weakens their treatment adherence. In the short term, resignation might alleviate distress, but in the longer term, it may be connected with an exacerbation of psychological distress and perpetuation of emotional issues (49). Studies have shown that resignation accelerates the decline of immune function by regulating the neuroendocrine mechanism, leading to continuous activation of the HPA axis and creating a vicious circle of physiological and psychological problems (50). For gastric cancer patients undergoing chemotherapy with confrontation-oriented, healthcare professionals can provide personalized education, such as active visualization to explain tumor shrinkage, and encourage patients to share their successful anti-cancer experiences (51). For individuals with avoidance and resignation-oriented approach, self-disclosure intervention program can be an effective treatment option (52). Through open communication, it is possible to assist people in reassessing their experience of sickness, which will help them rebuild their sense of control over the illness, promoting them to choose confrontation.

#### 4.2.4 Implications of self-transcendence theory for this study

The level of self-transcendence is the core concept of the theory. In this study, the illness perception, hope levels, coping modes, and health status awareness of gastric cancer patients undergoing chemotherapy align with the dimension of intrapersonal deepening and interpersonal expansion (25). Illness perception catalyzes intrapersonal deepening, triggering self-reflection and redefining life meaning through cognitive reframing, while motivating patients to seek external support. Hope levels function as the dynamic bridge between internal and external growth, enabling transcendence of current adversities through belief reinforcement, thereby fostering proactive coping strategies. This completes the synergistic practice of internal and external interactions. Self-rated health status is the ultimate manifestation of self-transcendence. Internally, it regards health as a state of physical and mental balance, rather than the absence of physical illness. Externally, it involves practicing a healthy lifestyle and maintaining an optimistic mindset to cope with illness. Guided by self-transcendence theory, the self-transcendence level of gastric cancer patients undergoing chemotherapy can be improved, helping them reconstruct their life meaning, increase treatment adherence, reduce symptom burden (53), and ultimately elevate quality of life through psychosocial-spiritual synergy.

## 5 Conclusion

This study employed an integrated analytical approach that combined random forest modeling and LASSO regression to rank variable importance and identify key determinants of self-transcendence, followed by multivariate stepwise linear regression. The findings revealed that self-transcendence levels among gastric cancer patients undergoing chemotherapy were predominantly low-to-moderate, significantly influenced by illness perception, hope levels, coping modes, and self-rated health status. This integrated analytical approach enhances the accuracy of the research findings, providing critical insights for clinicians to identify populations at risk for diminished self-transcendence and design targeted interventions based on the key determinants.

## 6 Limitations and future directions

Notable limitations include geographical restrictions, as samples were drawn from a single tertiary A hospital in Wuxi, China, and convenience sampling was employed. Consequently, the sample characteristics may not be representative of all gastric cancer patients, thereby limiting the applicability of the findings to broader or more diverse populations. In addition, the measurement results of scales may be subject to subjective bias and may be influenced by the emotional state of the subjects. Future studies should be conducted in a multi-center, large-scale setting, incorporating objective outcomes as endpoints to reduce bias, and using nationally representative cohort samples to validate and expand these conclusions.

## Data Availability

The original contributions presented in the study are included in the article/supplementary material. Further inquiries can be directed to the corresponding author.
