# Correction: Analysis of self-transcendence status and influencing factors in gastric cancer patients undergoing chemotherapy: a random forest model-based study

**DOI:** 10.3389/fpsyt.2025.1716981

**Published:** 2025-10-29

**Authors:** Qin Wang, Guoqin Ren, Li Sun, Xumiao Zhang, Hongxia Hua, Yanglin Gu

**Affiliations:** ^1^ Wuxi School of Medicine, Jiangnan University, Wuxi, China; ^2^ Department Public Health, Jiangnan University Medical Center, Wuxi, China; ^3^ Department Oncology, Jiangnan University Medical Center, Wuxi, China; ^4^ Department Orthopedic, Jiangnan University Medical Center, Wuxi, China

**Keywords:** gastric cancer, chemotherapy, self-transcendence, random forest model, influencing factors, artificial intelligence

In the affiliations, “Jiangnan University Medical Center” was erroneously given as “Organization Central Hospital of Jiangnan University”.

The original version of this article has been updated.

